# Clinical efficacy of anti‐amyloid antibodies in apolipoprotein E ε4 homozygotes: A Bayesian reanalysis of lecanemab and donanemab phase 3 results

**DOI:** 10.1002/trc2.70083

**Published:** 2025-04-09

**Authors:** Stefan Teipel, Yi Tang, Ara Khachaturian

**Affiliations:** ^1^ German Center of Neurodegenerative Diseases (DZNE), Rostock/Greifswald Rostock Germany; ^2^ Department of Psychosomatic Medicine University Medicine Rostock Rostock Germany; ^3^ Department of Neurology Capital Medical University Beijing China; ^4^ Brain Watch Coalition Rockville Maryland USA

**Keywords:** Alzheimer's disease, ApoE ε4, Bayesian reanalysis, CDR‐SB, clarity, TRAILBLAZER‐ALZ2

## Abstract

**INTRODUCTION:**

We aimed to determine the clinical efficacy of treating apolipoprotein E (ApoE) ε4 homozygotes with recently approved anti‐amyloid antibodies.

**METHODS:**

Data were derived from supplementary analyses in the regulatory studies Clarity (lecanemab) and TRAILBLAZER‐ALZ2 (donanemab). We used Bayesian reanalysis with an independent t‐statistic to determine evidence for or against an effect of antibody treatment on Clinical Dementia Rating scale Sum of Boxes (CDR‐SB) in ApoE ε4 homozygotes, and a Bayesian random‐effect meta‐analysis to determine the effect size.

**RESULTS:**

The Bayesian reanalysis showed moderate evidence of no effect for both antibodies. For donanemab and lecanemab, the odds of no difference in treatment effect were nearly three times greater than the odds of a difference. The meta‐analysis revealed a small effect of −0.06 CDR‐SB points in favor of treatment with a moderate heterogeneity estimate. The Bayes factor was 0.26, indicating that the absence of an effect was almost four times more likely than the presence of an effect.

**DISCUSSION:**

The most likely explanation for our results is the lack of a treatment effect for lecanemab and donanemab in ApoE *ε*4 homozygotes. This could reflect inadequate exposure to the antibody due to more severe side effects, subsequent treatment interruptions, and lower dosing or a biologically driven lack of efficacy in a genetically determined disease. Our results support the view that excluding these cases from treatment is justifiable because of the higher risk of side effects and the lack of clinical efficacy.

**Highlights:**

- Lecanemab and donanemab were clinically ineffective in ApoE ε4 homozygotes.
- ApoE ε4 homozygotes should not receive these treatments due to inefficacy.
- Future trials should adopt Bayesian analysis strategies.
- Bayesian analysis provides evidence for or against treatment effects.
- Bayesian inference provides clinically interpretable results.

## 1 INTRODUCTION

The decision of the UK Medicines and Healthcare products Regulatory Agency (MHRA) on August 22, 2024, to approve a product license for lecanemab “to treat adults in the early stages of Alzheimer's disease (AD) who have one or no copies of the apolipoprotein Ε4 gene” and the recommendation of the Committee for Medicinal Products for Human Use (CHMP) of the European Medicines Agency (EMA) on November 14, 2024, to grant a marketing authorization for lecanemab only “in apolipoprotein E (ApoE) ε4 non‐carriers or heterozygotes” has once again drawn attention to the question of anti‐amyloid antibody treatment of homozygote carriers of the ApoE ε4 genotype. In the phase 3 data of the antibodies donanemab and lecanemab ApoE ε4 homozygotes had a significantly higher risk of amyloid‐related imaging abnormalities (ARIA) and related symptoms compared to heterozygotes and non‐carriers.^1^, ^2^ In prespecified secondary analyses, lecanemab showed a trend effect in favor of placebo and donanemab showed a trend effect in favor of treatment in the Apo Ε4 homozygote groups, with 95% confidence intervals (CIs) including the null value in both cohorts. The Appropriate Use Recommendations for lecanemab “recommend APOE genotyping of all treatment candidates before initiating lecanemab therapy”,^3^ but, consistent with the Federal Drug Administration (FDA)^4^ and inconsistent with the UK MHRA and EMA's CHMP recommendations, do not exclude ApoE ε4 homozygotes from treatment.

Here, we addressed the question whether donanemab and lecanemab have a different clinical effect in ApoΕ4 homozygotes on the Clinical Dementia Rating—Sum of Boxes (CDR‐SB)^5^ as an estimate of clinical effect size. We used a Bayesian reanalysis approach^6^ to directly quantify clinical efficacy of both antibodies and a difference in clinical efficacy between the two antibodies in ApoE ε4 homozygotes. A low probability of benefit would support the decisions of the MHRA and the EMA's CHMP not only on the basis of adverse effects, but also on the basis of lack of clinical efficacy.

## 2 METHODS

### 2.1 Data source

We analyzed data from the supplementary material of the Clarity^2^ and the TRAILBLAZER‐ALZ2^1^ trials. Specifically, we determined the mean differences (MDs), their 95% confidence intervals and the number of cases in the placebo and treatment groups for the effect of donanemab and lecanemab, respectively, on the CDR‐SB and for the difference in effect between the two antibodies in Apo Ε4 homozygotes. The data were obtained from the supplementary sections of the TRAILBLAZER‐ALZ2^1^ and Clarity^2^ publications, specifically from page 35, “eFigure 9D” of “Supplement 3. eMethods and eResults” of TRAILBLAZER‐ALZ2,^1^ and page 17, “Figure S1”, of the “Supplementary Appendix” of Clarity.^2^ The phase 2 trial of donanemab, TRAILBLAZER‐ALZ,^7^ and the phase 2 trial of lecanemab, BAN2401‐G000‐201,^8^ did not report data on treatment effects stratified by ApoE4 genotype.

We calculated the standard error (SE) of the effect as SE = |upper threshold 95% confidence interval—lower threshold 95% confidence interval|/2* t(df, *p* < 0.025),^9^ with t representing the Student's t distribution with df (degrees of freedom) equal to the total number of cases. With df > 120, as was the case in our analysis with > 200 cases per comparison, t(df, *p* < 0.025) approximates z(*p* < 0.025), the standard normal distribution (see section 6.5.2.2 of the Cochrane Training Manual, https://training.cochrane.org/handbook/current/chapter‐06#section‐6‐5‐2‐2).

The SE of the MD of the effects between the antibodies was calculated as:

$\;SE_{diff}=\sqrt{SE_{lec}^{2}+SE_{don}^{2}}$, that is, the square root of the sum of the squared SEs of the treatment effects.

The mean effect estimate T was calculated as the MD between placebo and treatment groups and the MD of effects, respectively, divided by the corresponding SE, T = MD/SE.

### 2.2 Statistical analysis

We conducted two analyses, a Bayesian reanalysis and a Bayesian random effect meta‐analysis.

The Bayesian reanalysis followed an approach suggested by Costa and colleagues^6^ using Bayesian independent samples T tests in JASP, version 0.18.3, on the effect size T based on the differences of the CDR‐SB and the estimate of the SE. We calculated the Bayes factor (BF) in favor of the two‐sided alternative hypothesis of the presence of a treatment effect (BF_10_). According to Bayesian analysis reporting guidelines,^10^ a BF_10_ between 3 and 10 indicates a moderate, a BF_10_ between 10 and 30 indicates a strong, a BF_10_ between 30 and 100 indicates a very strong, and a BF_10_ > 100 indicates an extreme level of evidence in favour of the alternative model over the null model. Equivalently, if BF_10_ is below 1/3, 1/10, 1/30, 1/100, it indicates a moderate, strong, very strong, or extreme level of evidence, respectively, in favour of the null over the alternative hypothesis.

We conducted the following comparisons: lecanemab versus placebo, donanemab versus placebo, and effect of donanemab versus effect of lecanemab. We used BF robustness checks by running the analyses over a wide range of scales of the Cauchy prior, between 0.01 and 1.5, with the default Cauchy prior having a scale of 0.707. Following a discussion about Bayesian reanalysis of clinical trials,^11^, ^12^ we also adopted the “adversarial perspective”.^11^ An “adversarial prior” is a prior that assumes an effect that a “scientific adversary” would assume. Citing from,^11^ page 488: “By using an adversarial perspective, a Bayesian reanalysis can be used to specifically assess whether the available data are strong enough to forge consensus among reasonable and well‐intentioned experts. If the point estimate of a trial indicates benefit, regardless of the *p* value, consensus can arise only if the data are strong enough to convince those with reasonable pessimistic prior expectations. Conversely, if the trial results indicate harm or a negligible effect, it is the position of the reasonable optimists that requires careful evaluation.” It was the latter, the optimist's assumption that donanemab also works in ApoE ε4 homozygotes, that we wanted to test with the “adversarial prior”.

For this purpose, we used robust t‐distributions with three df for the adversarial priors. For the effect of donanemab, we derived the effect estimate and its SE from the previous phase 2 trial (TRAILBLAZER‐ALZ),^7^ reflecting the optimistic view that the effect in ApoE ε4 homozygotes is equivalent to the overall effect in the previous phase 2 trial. The effect in TRAILBLAZER‐ALZ was −0.36 [95% confidence interval −0.83–0.12] CDR‐SB points.^7^ This gives a SE of |0.12–−0.83|/(2*1.96) = 0.24 CDR‐SB points and a scale parameter of $SE*\sqrt{\frac{df}{df-2}}$ = 0.42.

For the difference between donanemab and lecanemab, we could not use estimates from previous studies. Therefore, to reflect the optimistic view, we used the observed effect size (Cohen's d) of −0.15 from the current analysis and the SE of 0.43. From this, we derived the t‐distribution with three df, centered at −0.15 with a scale parameter of $SE*\sqrt{\frac{df}{df-2}}$ = 0.745.

In addition, we conducted a Bayesian random‐effects meta‐analysis with the library “bayesmeta” in R^13^ to determine estimates for the effects of treatment on CDR‐SB in the ApoE ε4 homozygotes. This analysis provided us with following three measures together with their 95% credible intervals:

1. The mean effect size in the units of the primary endpoint, here score points of the CDR‐SB;
2. The prediction value, indicating the expected effect for a new study using the same or similar antibodies, based on the posterior predictive distribution;
3. The heterogeneity parameter tau (τ), quantifying the amount of between‐study variation that is not explained by the fixed effect. The parameter τ equals the variance of the random effect minus the within‐study sampling variance so that, if τ increases, the variance of the random effect also increases, which means that the random effect estimate becomes less precise.

**RESEARCH IN CONTEXT**

1. **Systematic review**: We reviewed the literature using pubmed, meeting abstracts and presentations. Several meta‐analyses have examined the efficacy of anti‐amyloid antibodies in Alzheimer's disease (AD), but none have quantified the evidence for or against a clinical effect in ApoE ε4 homozygotes.
2. **Interpretation**: The results suggest moderate evidence against a clinical benefit of lecenamab and donanemab treatment in ApoE ε4 homozygotes.
3. **Future directions**: The results support the exclusion of ApoE ε4 homozygotes from current anti‐amyloid treatment due to side effects and lack of clinical efficacy. Bayesian reanalysis is only the second‐best option. Therefore, future trials should incorporate Bayesian inference into the study design as a primary or secondary analysis strategy to provide clinically interpretable evidence for or against treatment effects.

In addition, we determined the BF_10_ for an effect of treatment. For the effect estimate we chose a weakly informed normally distributed prior with mean 0 and standard deviation (SD) of 1. The prior for the heterogeneity parameter τ was chosen as a weakly informed half‐normal distribution with a SD of 0.5, consistent with a previous systematic review of heterogeneity estimates across a large range of meta‐analyses from the Cochrane library.^14^ In a sensitivity analysis, we determined effects when using an uninformative Jeffreys prior for heterogeneity, and a value of four for the prior of the SD of the effect estimate. Third, to account for imprecision in deriving SE estimates from the graphs, we conducted a sensitivity analysis in which we repeated the analyses with 0.2 times the SE added to the SEs' estimates.

## 3 RESULTS

**FIGURE 1:**
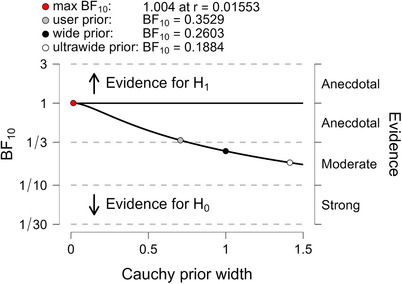
BF robustness check of the Bayesian t‐test for donanemab. Estimates of the BF in favor of the alternative hypothesis (BF_10_) depending on the choice of the width of the Cauchy prior. The default prior width is indicated as a gray dot, a wide prior as black dot, an ultrawide prior as a white dot, and the prior scale yielding the maximum BF_10_ value as a red dot. BF, Bayes factor.

**FIGURE 2:**
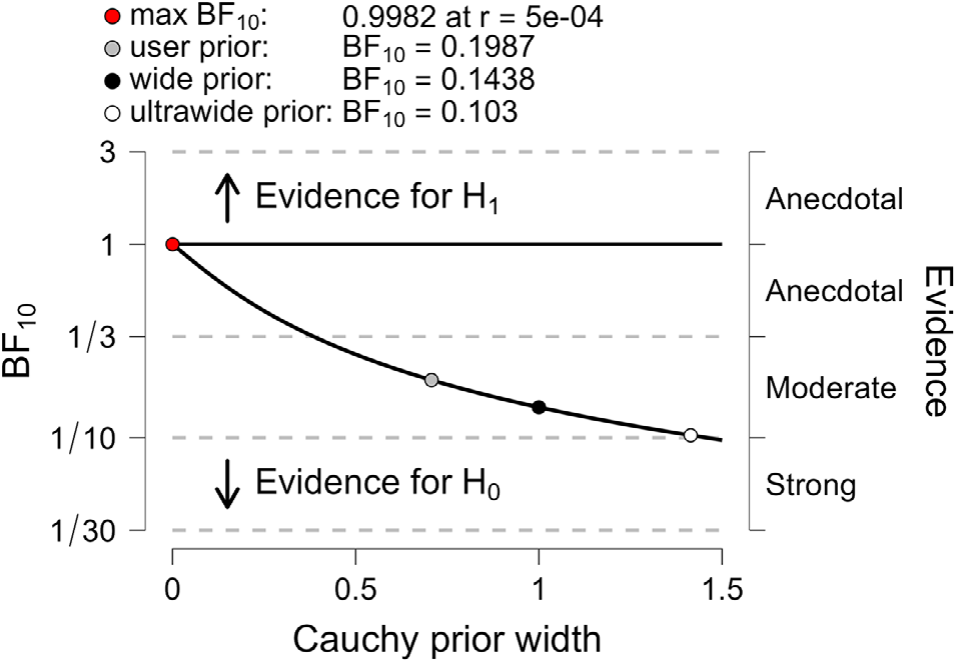
BF robustness check of the Bayesian t‐test for lecanemab. Estimates of the BF in favor of the alternative hypothesis (BF_10_) depending on the choice of the width of the Cauchy prior. The default prior width is indicated as a gray dot, a wide prior as black dot, an ultrawide prior as a white dot, and the prior scale yielding the maximum BF_10_ value as a red dot. BF, Bayes factor.

**FIGURE 3:**
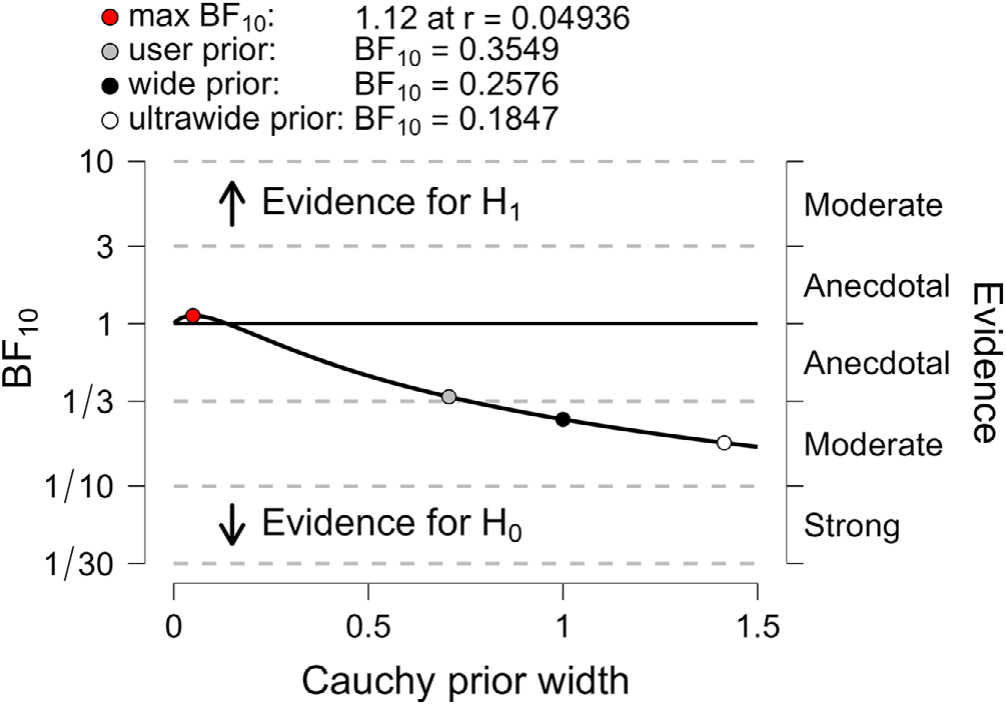
BF robustness check of the Bayesian t‐test for the difference between donanemab and lecanemab. Estimates of the BF in favor of the alternative hypothesis (BF_10_) depending on the choice of the width of the Cauchy prior. The default prior width is indicated as a gray dot, a wide prior as black dot, an ultrawide prior as a white dot, and the prior scale yielding the maximum BF_10_ value as a red dot. BF, Bayes factor.

Estimates for MDs, SEs, T value, and Cohen's d are shown in Table 1. The Bayesian reanalysis of donanemab showed a BF_10_ = 0.353, suggesting that the absence of an effect of treatment was almost three times more likely than the presence of an effect. For lecanemab, the BF_10_ was 0.20, suggesting that the absence of an effect of treatment was five times more likely than the presence of an effect. For the difference of donanemab and lecanemab, BF_10_ was 0.355, suggesting that the absence of a difference in the treatment effects of donanemab and lecanemab was almost three times more likely than the presence of such an effect. When we checked the robustness of the BF_10_, we found for all three comparisons that even with an unrealistically narrow scale of the Cauchy prior, the maximum BF_10_ was only around 1, indicating inconclusive evidence. The evidence for no effect increased with the width of the prior (Figures 1, 2, 3). For the “adversarial perspective”,^11^ we chose priors in favor of a treatment effect to reflect the prior belief of a scientific “adversary” who would be optimistic about a treatment effect in ApoE ε4 homozygotes. For donanemab, with selected a t‐distributed prior with three df centered on a mean effect of −0.36, corresponding to the overall effect in the phase 2 TRAILBLAZER‐ALZ trial,^7^ and a scale parameter of 0.42. The resulting BF_10_ was 0.64, indicating inconclusive evidence of no effect. For the difference between donanemab and lecanemab, with a t‐distributed prior centered at an effect size of −0.15, a scale parameter of 0.745 and three df, the BF_10_ was 0.41, that is, indicating inconclusive evidence of no effect.

**TABLE 1:** Estimates for effects on CDR‐SB

Study	MD	SE	N	T	SD	Cohen's d
TRAILBLAZER‐ALZ2^a^	−0.41	0.30	123/97^c^	−1.36	2.22	−0.19
Clarity^b^	0.28	0.31	132/136^c^	0.91	2.51	0.11
donanemab versus lecanemab	−0.69	0.43	220/268^d^	−1.61	4.73	−0.15

Abbreviations: MD, mean difference; SD, standard deviation of effect; SE, standard error of MD.

^a^ donanemab versus placebo.

^b^ lecanemab versus placebo.

^c^ number of cases (placebo/treatment).

^d^ number of cases (donanemab/lecanemab).

**FIGURE 4:**
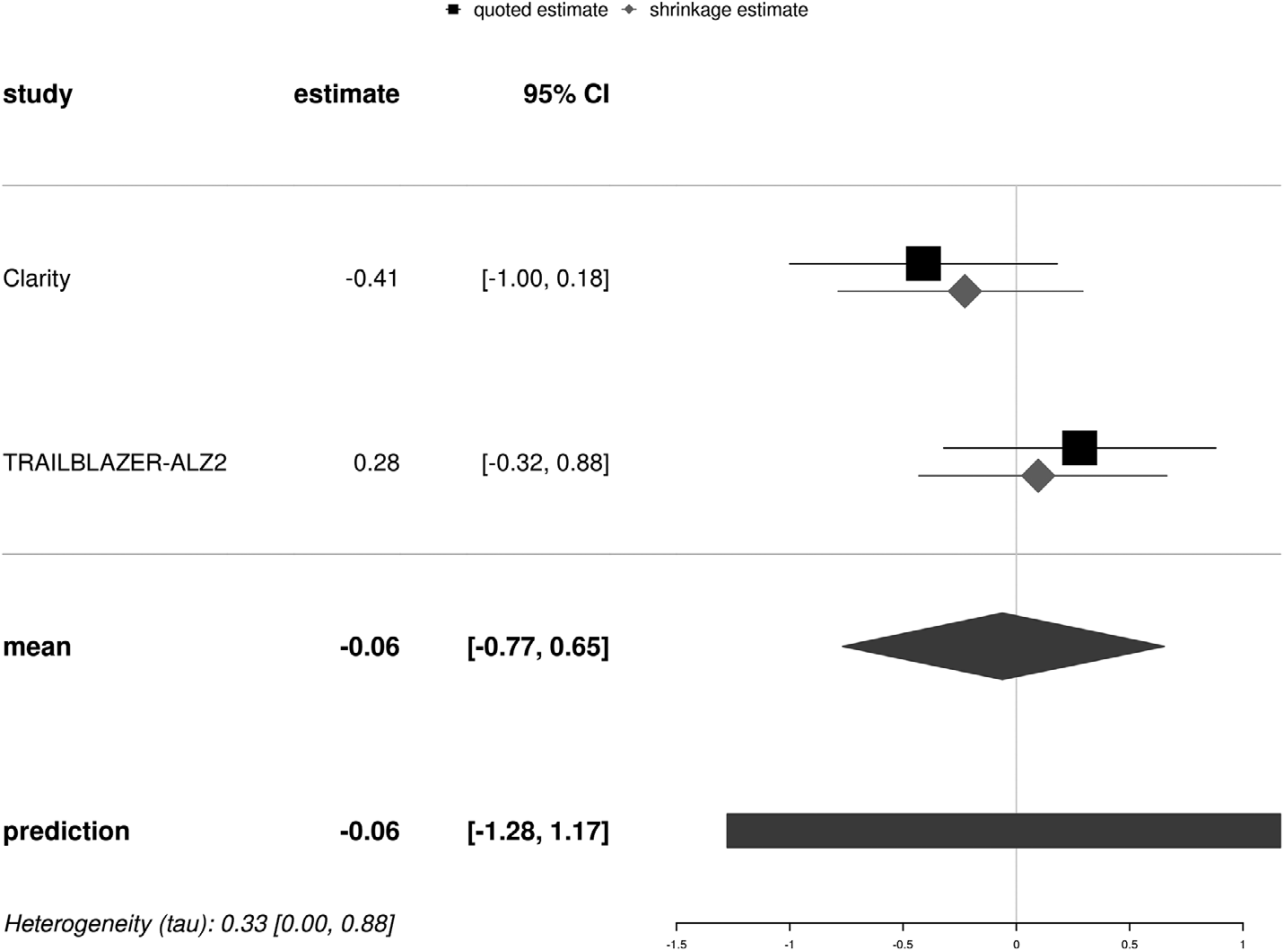
Forest plot. The forest plot features the direct and indirect estimates of treatment effects across the cohorts. The direct or quoted estimates are the parameter estimates based only on the effects in the particular study, while the indirect or shrinkage estimates are the estimates that shrunk to the mean of all studies, taking into account the information from all other studies for the single estimate. Negative numbers favor treatment. Estimates are based on Bayesian random effect meta‐analysis models with weakly informative priors (normal (mean = 0, standard deviation = 1)) and heterogeneity priors (half‐normal (scale = 0.5)). 95% CI, credible interval.

The results of the meta‐analysis of Clarity and TRAILBLAZER‐ALZ2 in ApoE ε4 homozygotes are shown in Figure 4. We found a small overall effect of −0.06 CDR‐SB points in favor of treatment with a wide 95% credible interval and a moderate heterogeneity estimate. Consistently, the predicted effects from the posterior predictive distribution were widely and nearly symmetrically distributed around the null. The BF_10_ for an effect was 0.26, indicating that the absence of an effect was almost four times more likely than the presence of an effect. The results were preserved in the sensitivity analysis with a 0.2 times higher estimate of the SEs of the MDs (Figure S1). Using an uninformative Jeffrey's prior moderately increased the heterogeneity estimate from 0.33 [95% credible interval 0–0.88] to 0.59 [95% credible interval 0.01–2.76] CDR‐SB points (Figure S2). Using a higher SD estimate of four had little effect on the effect size estimate (Figure S3).

## 4 DISCUSSION

Here, we determined the effects of treatment with donanemab and lecanemab on CDR‐SB and the difference in treatment effects between both compounds in the ApoΕ4 homozygote subgroups of the TRAILBLAZER‐ALZ2 and Clarity trials.^1^, ^2^ The absence of a difference in treatment effects for donanemab compared with lecanemab was three times more likely than the presence of such an effect. The absence of a treatment effect for each of the two antibodies, donanemab and lecanemab, was three to five times more likely than the presence of such an effect in the Bayesian reanalysis. In a Bayesian meta‐analysis across both trials, we found a very small effect estimate of −0.06 CSR‐SB points in favor of treatment; the absence of a treatment effect was almost four times more likely than the presence of a treatment effect.

These data suggest that neither a trend in favor of treatment nor a trend in favor of placebo indicates a qualitative difference in effect between donanemab and lecanemab in ApoE ε4 homozygotes and that both drugs had no effect rather than an effect in these cases. In conclusion, neither drug had qualitatively different treatment effects on CDR‐SB in ApoΕ4 homozygote carriers, and the absence of a treatment effect is the most plausible interpretation for both drugs.

Of note, this statistical analysis does not allow any inference on underlying causes. Thus, our results do not exclude the interpretation of the data that the lack of effect in ApoΕ4 homozygotes was due to insufficient exposure to the drug, because ApoΕ ε4 cases had a higher risk of ARIA and therefore had more frequent treatment interruptions and lower dosages.^15^ The results are, however, equally compatible with the assumption of lower biological efficacy in this specific group of patients that has a genetically driven disease.^16^ As an aside, our analysis illustrates the advantage of a Bayesian analysis in providing a quantitative estimate of the level of evidence for the effect of interest that is directly interpretable and intuitive.

There are several limitations of our study. First, we had no access to the original data but derived the effect size estimates from the numbers and figures in the supplements of the two studies.^1^, ^2^ Second, BF estimates are sensitive to the choice of the priors. Therefore, we conducted sensitivity analyses to check the robustness of the BFs. The results of these sensitivity analyses showed that the conclusions would hold even with extreme choices of prior scales. The data were even strong enough to overcome priors that reflected a very optimistic view of treatment effects, following the “adversarial perspective”.^11^ Our analysis cannot resolve the cause of the lack of effect, whether it is a lower dose of antibodies due to a higher rate of adverse events, or a lower biological effect in people with this genotype, or both. Third, it could be argued that the concepts underlying the original design and analysis of the studies, and the current reanalysis of the data are quite different. The trials were designed and analyzed on the basis of the frequentist framework, with the goal of controlling the error rate of the experiment in the long run. The Bayesian analysis asks the question how likely it is that an effect exists, given the observed data, and how large the effect is. In short, frequentist analysis asks how likely the data or more extreme data are if the null hypothesis is true, while Bayesian analysis asks how likely the null hypothesis or the alternative hypothesis is given the data. Obviously, the two conditional probabilities cannot simply be transposed, that is, a low probability of the data given the null hypothesis is true does not necessarily mean a low probability of the null hypothesis given the data, and vice versa. Finally, the meta‐analysis is based on only two trials, which particularly limits the estimation of heterogeneity. Nevertheless, we decided to perform a meta‐analysis to provide an estimate of the effect size based on the limited data currently available. The results of the Bayesian reanalysis and the meta‐analysis are complementary, because the Bayesian reanalysis focuses mainly on the evidence for and against an effect based on the BF, whereas the meta‐analysis focuses mainly on the size of the effect and its heterogeneity.

The lack of treatment effect for both lecanemab and donanemab in ApoE ε4 homozygotes is the most likely explanation for our findings. This lack of effect may be due to insufficient exposure to the antibody as a consequence of higher side effects and subsequent treatment interruptions and lower doses,^15^ or to a biological lack of efficacy in a genetically determined disease^16^ or both. The CHMP of the EMA has recommended granting the marketing authorization for Leqembi (lecanemab) in November 18, 2024, in ApoE ε4 non‐carriers or “heterozygotes”, after initial refusal of recommending a marketing authorization in July 2024.^17^ One concern in the CHMP's first and now also in the final decision was the higher risk of side effects, particularly of ARIAs, in ApoE ε4 homozygotes. Our results support the notion that excluding these cases from treatment is ethically justifiable not only because of the higher risk of side effects, but also because of the lack of clinical efficacy. The exemption of ApoE ε4 homozygotes in the UK MHRA decision and the EMA's CHMP recommendation does not withhold a clinically effective treatment from this group of patients.

## CONFLICT OF INTEREST STATEMENT

S.T. was member of Advisory Boards of Eisai, Lilly, GE Healthcare, and Biogen. He was member of the Data Safety and Monitoring Board of the ENVISION study (Biogen). Y.T. and A.K. report no conflicts of interest. Author disclosures are available in the Supporting Information.

## CONSENT STATEMENT

The study presents a reanalysis of published data, therefore, consent of human subjects was not necessary.

## Supporting information



Supporting Information

Supporting Information

## References

[1] Sims JR , Zimmer JA , Evans CD , et al. Donanemab in early symptomatic Alzheimer disease: the TRAILBLAZER‐ALZ 2 Randomized Clinical Trial. J Am Med Assoc JAMA. 2023;330(6):512‐527. 10.1001/jama.2023.13239 PMC10352931 37459141

[2] van Dyck CH , Swanson CJ , Aisen P , et al. Lecanemab in early Alzheimer's disease. N Engl J Med. 2023;388:9‐21. 36449413 10.1056/NEJMoa2212948

[3] Cummings J , Apostolova L , Rabinovici GD , et al. Lecanemab: appropriate use recommendations. J Prev Alzheimers Dis. 2023;10:362‐377. 37357276 10.14283/jpad.2023.30 PMC10313141

[4] FDA . Lequembi. 2023. https://www.accessdata.fda.gov/drugsatfda_docs/label/2023/761269s000lbl.pdf

[5] Wessels AM , Dowsett SA , Sims JR . Detecting treatment group differences in Alzheimer's disease clinical trials: a comparison of Alzheimer's Disease Assessment Scale—Cognitive Subscale (ADAS‐Cog) and the Clinical Dementia Rating—Sum of Boxes (CDR‐SB). J Prev Alzheimers Dis. 2018;5:15‐20.29405227 10.14283/jpad.2018.2

[6] Costa T , Premi E , Liloia D , Cauda F , Manuello J . Unleashing the power of Bayesian re‐analysis: enhancing insights into lecanemab (Clarity AD) Phase III trial through informed t‐test. J Alzheimers Dis. 2023;95:1059‐1065. 37638445 10.3233/JAD-230589

[7] Mintun MA , Lo AC , Duggan Evans C , et al. Donanemab in early Alzheimer's disease. N Engl J Med. 2021;384:1691‐1704. 33720637 10.1056/NEJMoa2100708

[8] Swanson CJ , Zhang Y , Dhadda S , et al. A randomized, double‐blind, phase 2b proof‐of‐concept clinical trial in early Alzheimer's disease with lecanemab, an anti‐Aβ protofibril antibody. Alzheimers Res Ther. 2021;13:80. 33865446 10.1186/s13195-021-00813-8 PMC8053280

[9] Higgins JPT , Cochrane Collaboration . Cochrane Handbook for Systematic Reviews of Interventions. 2nd ed. Wiley‐Blackwell; 2020.

[10] van Doorn J , van den Bergh D , Bohm U , et al. The JASP guidelines for conducting and reporting a Bayesian analysis. Psychon Bull Rev. 2021;28:813‐826. 33037582 10.3758/s13423-020-01798-5 PMC8219590

[11] de Grooth HJ , Cremer OL . Bayes and the evidence base: reanalyzing trials using many priors does not contribute to consensus. Am J Respir Crit Care Med. 2024;209:483‐484. 37922492 10.1164/rccm.202308-1455VP PMC10919112

[12] Goligher EC , Harhay MO . What is the point of Bayesian analysis? Am J Respir Crit Care Med. 2024;209:485‐487. 37922491 10.1164/rccm.202310-1757VP PMC10919113

[13] Rover C . Bayesian random‐effects meta‐analysis using the bayesmeta R package. J Stat Softw. 2020;93:1‐51.

[14] Rhodes KM , Turner RM , Higgins JPT . Predictive distributions were developed for the extent of heterogeneity in meta‐analyses of continuous outcome data. J Clin Epidemiol. 2015;68:52‐60. 25304503 10.1016/j.jclinepi.2014.08.012 PMC4270451

[15] Evans CD , Zimmer JA , Wessels AM , et al. Efficacy of Donanemab by APOE ε4 carrier status in TRAILBLAZER‐ALZ 2, a Phase 3 randomized Clinical Trial in Early Symptomatic Alzheimer's Disease. In: Clinical Trials on Alzheimer's Disease (CTAD)—16th Annual Conference, Boston, Massachusetts, USA . 2023.

[16] Fortea J , Pegueroles J , Alcolea D , et al. APOE4 homozygosity represents a distinct genetic form of Alzheimer's disease. Nat Med. 2024;30:1284‐1291. 38710950 10.1038/s41591-024-02931-w PMC13310155

[17] European Medicinal Agency (EMA) . The European Medicines Agency has recommended the refusal of the marketing authorisation for Leqembi. https://www.ema.europa.eu/en/medicines/human/EPAR/leqembi#:~:text=The%20European%20Medicines%20Agency%20has%20recommended%20the%20refusal,Agency%20issued%20its%20opinion%20on%2025%20July%202024.2024

