# Supplementary material for: Clinical efficacy of anti‐amyloid antibodies in apolipoprotein E ε4 homozygotes: A Bayesian reanalysis of lecanemab and donanemab phase 3 results
Source: Alzheimers Dement (N Y). 2025 Apr 9;11(2):e70083. doi: 10.1002/trc2.70083 (PMC11982174; doi:10.1002/trc2.70083)
Supplement: Supplementary file 1 — Supporting Information [file TRC2-11-e70083-s002.docx]

**Supplementary Figure 1: Forest plot of sensitivity analysis –higher estimates of standard errors**


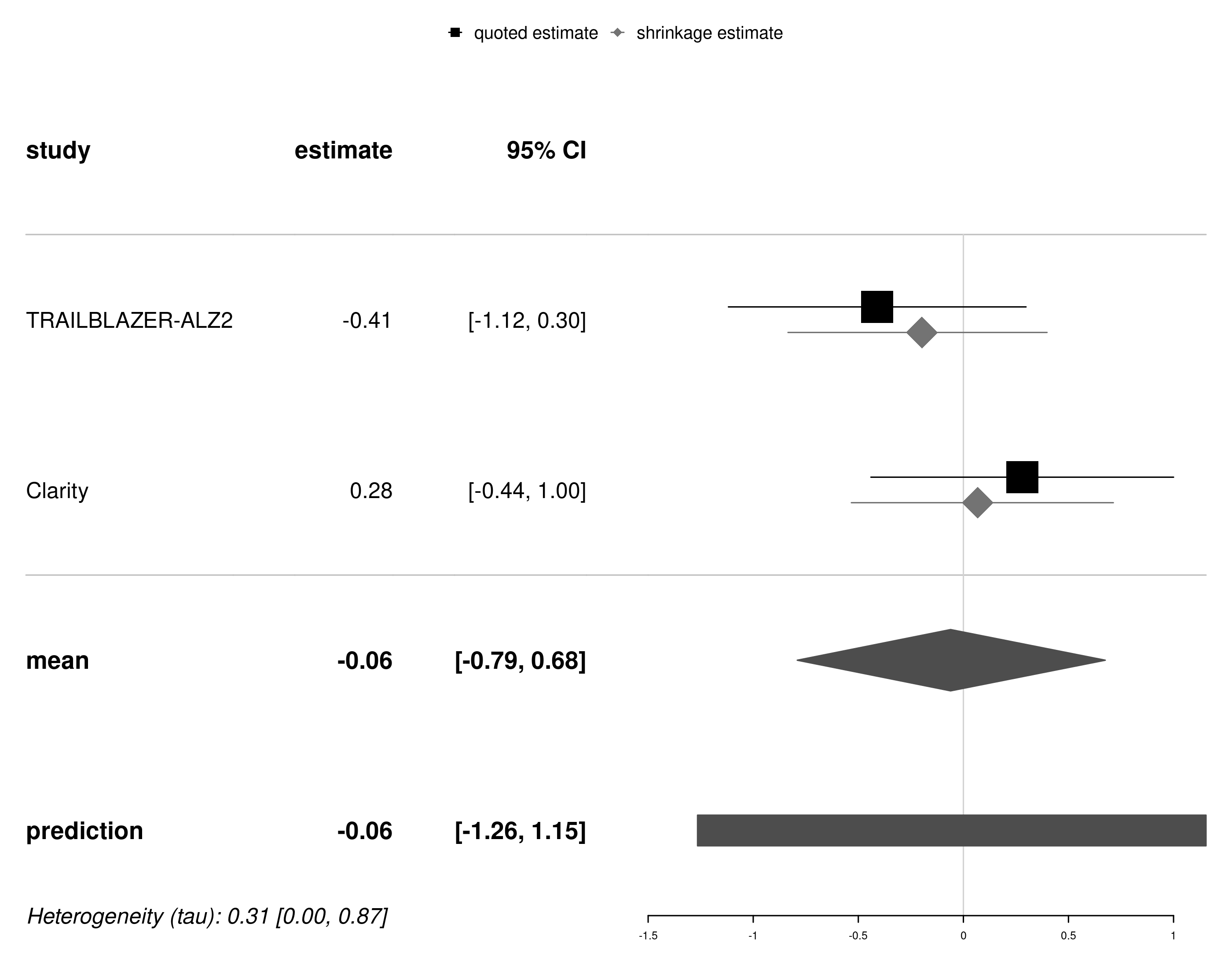


The forest plot features the direct and indirect estimates of treatment effects across the cohorts. The direct or quoted estimates are the parameter estimates based only on the effects in the particular study, while the indirect or shrinkage estimates are the estimates shrunk to the mean of all studies, taking into account the information from all other studies for the single estimate.

Negative numbers favor treatment.

Estimates are based on Bayesian random effect meta-analysis models with weakly informative priors (normal (mean= 0, standard deviation = 1)) and heterogeneity priors (halfnormal (scale = 0.5)), and a 0.2 times higher estimate of the standard error.

95% CI - 95% credible interval

**Supplementary Figure 2: Forest plot of sensitivity analysis – uninformative prior for the heterogeneity estimate**

**
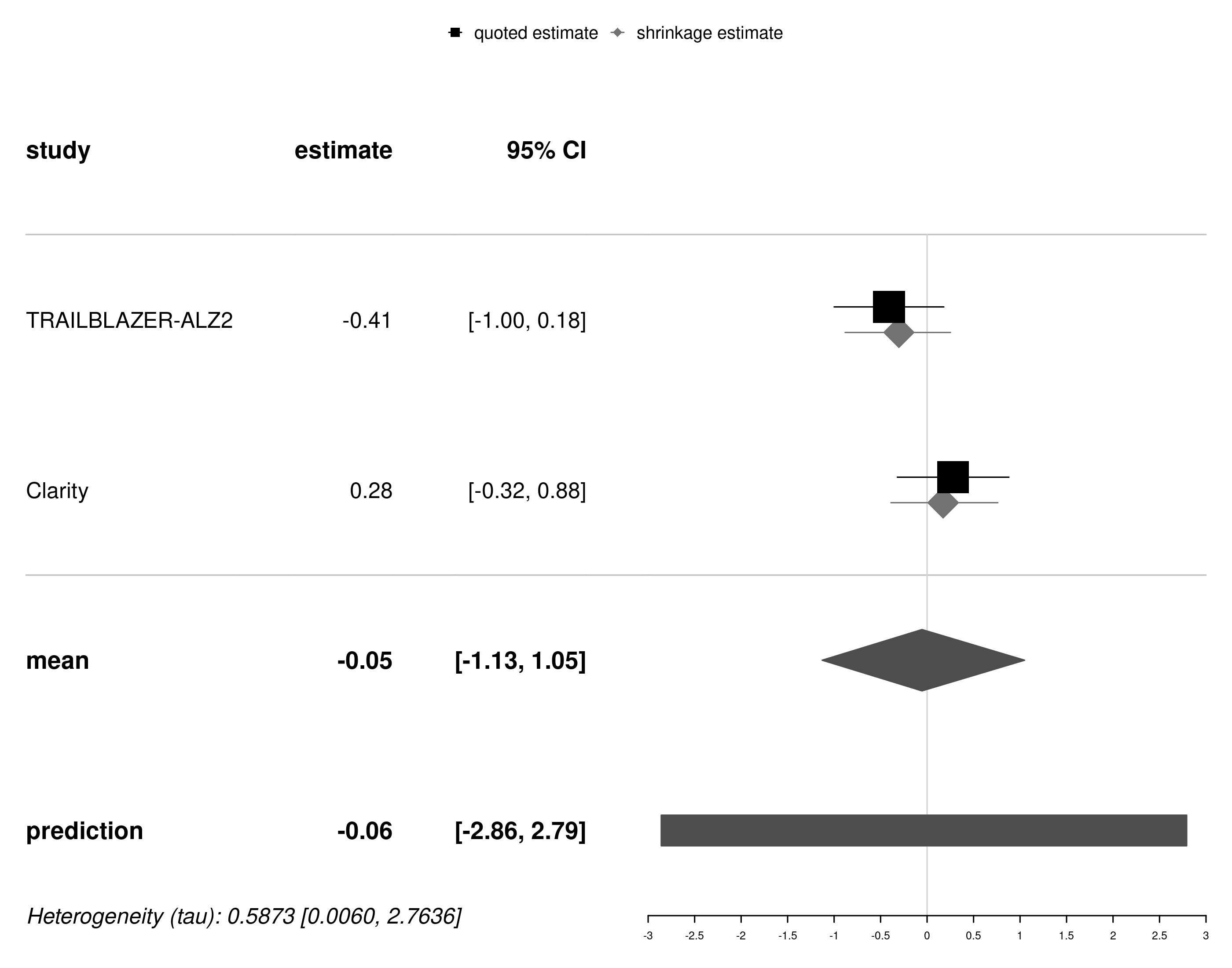
**

The forest plot features the direct and indirect estimates of treatment effects across the cohorts. The direct or quoted estimates are the parameter estimates based only on the effects in the particular study, while the indirect or shrinkage estimates are the estimates shrunk to the mean of all studies, taking into account the information from all other studies for the single estimate.

Negative numbers favor treatment.

Estimates are based on Bayesian random effect meta-analysis models with a weakly informative prior for the effect estimate (normal (mean= 0, standard deviation = 1)) and an uninformative heterogeneity prior (Jeffrey).

95% CI - 95% credible interval

**Supplementary Figure 3: Forest plot of sensitivity analysis – higher standard deviation of the prior of the effect estimate**

**
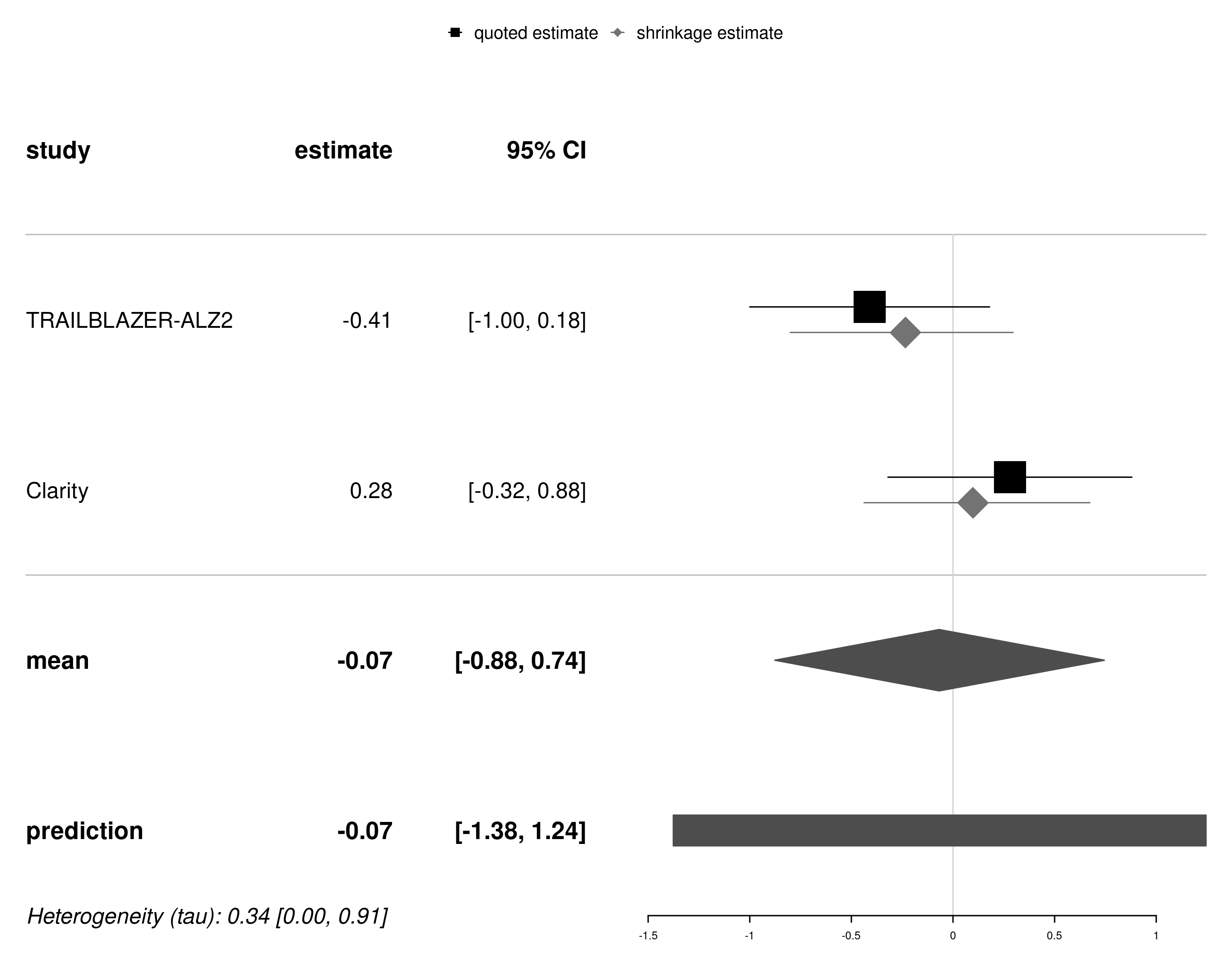
**

The forest plot features the direct and indirect estimates of treatment effects across the cohorts. The direct or quoted estimates are the parameter estimates based only on the effects in the particular study, while the indirect or shrinkage estimates are the estimates shrunk to the mean of all studies, taking into account the information from all other studies for the single estimate.

Negative numbers favor treatment.

Estimates are based on Bayesian random effect meta-analysis models with a very weakly informative prior for the effect estimate (normal (mean= 0, standard deviation = 4)) and a weakly informative heterogeneity prior (halfnormal (scale = 0.5)).

95% CI - 95% credible interval
